# Humor, transparency, and the management of distrust among business rivals: a case study of berthing meetings at the Port of Tema in Ghana

**DOI:** 10.1007/s40152-023-00298-1

**Published:** 2023-03-07

**Authors:** Martin Arvad Nicolaisen, Annette Skovsted Hansen

**Affiliations:** Global Studies, Aarhus University, Jens Chr. Skousvej 7, 4, 8000 Aarhus, Denmark

**Keywords:** Maritime, Port, Humor, Trust, Africa, Transparency

## Abstract

This article builds on rich empirical data following our unexpected discovery of a local practice to circumvent a stressful and counterproductive work environment due to distrust at the Port of Tema in Ghana. Using theoretical work on networks, trust, and humor, as well as extensive ethnographic fieldwork, we found that the humorous atmosphere at the regularly held physical berthing meetings fosters a sense of community, which enables competing professions, private companies, and public institutions to manage their mutual distrust. In an environment where trust among competitors is unrealistic, we argue that the objective of the performance of humor and transparency at the physical berthing meetings is the management of distrust rather than the creation of trust. The meetings have, gradually, grown to serve as a pragmatic local stakeholder adaptation to the challenges posed by universally perceived politicized, opaque, and corrupt business practices at the Port of Tema and beyond. In conclusion, we posit that our empirical findings allow us to identify the potential of and gaps in theories about trust and humor in understanding the dynamics of coping strategies among competitors in business settings that are characterized by unethical practices.

## Introduction

Many members of the port community at the Port of Tema, Ghana, continue to attend so-called berthing meetings in person as a way to increase transparency and manage distrust among colleagues and competitors in a business setting characterized by decades of corruption and other breaches to ethics. Our key finding is that humor serves to keep distrust at manageable levels by reducing stress and questioning the in-transparency prevalent in port related decision-making. This article, its topic, and questions are the unanticipated outcome of field research conducted between 2019 and 2022 for our research project on port efficiency, digitalization, and capacity development (PEPP) (https://projects.au.dk/pepp-ii). The combination of historical and anthropological approaches based on qualitative materials and social scientific theory ensure a diachronous and synchronous richness of material, which allows us to identify new promising lines of research into the interstices between trust and humor.

At the Port of Tema in Ghana, some 50 people meet twice a week to announce and confirm information already shared digitally about berth assignments for incoming vessels and work assignments for the stevedoring companies. Public and private sector employees of the local shipping industry attend the meetings that last 2–3 hours in a conference room at the port administration building. During the meetings, they mix formal business announcements with humorous discussions and the issuance of so-called funny fines. Our key questions are as follows: Why physical meetings continue to enjoy local support and how humor and other practices make it worthwhile for colleagues and competitors to meet in person, each time paying money out of their own pockets to do so? To answer these questions, this article proceeds along two lines of enquiry: One addresses the “why” by discussing “trust” in the context of the history of the Port of Tema and in literature on the recent history of the independent nation of Ghana. The other addresses the “how” by presenting findings from detailed and extensive ethnographic observations of the humor performed at the berthing meetings, and an analysis of the effects of the interactions.

In the “The Port of Tema and berthing meetings” section, we introduce the empirical setting of the Port of Tema in Ghana including an exploration of how challenges experienced over the past half a century have shaped the present needs for transparency and trust. Here, we also outline the function of the local berthing meetings. In the “Theory” section, we present the theories, with which we engage. In the “Methodology” section, we explain the methodology behind our data collection. This is followed by the “Findings: distrust in a politicized port business environment” section, where we present our findings concerning trust, and the “Findings: humor builds community” section with our findings concerning the significance of humor in building a sense of shared community among the different operational level port stakeholders. In the “Discussion” section, we combine the theories and our findings on trust and humor and discuss how humor at the berthing meetings enables a shared port user community on the operational level. In the “Conclusion: managing distrust” section, we conclude that the combination of humor and the collection of cash fines at the physical berthing meetings contribute to the management of distrust. Hereby, this article contributes to our understanding of how local adaptations to breaches of business ethics can improve daily work life.

## The Port of Tema and berthing meetings

Senior berthing meeting participants explain the original establishment and the continued need for berthing meetings as a collective response to negative factors grounded in the history of the Port of Tema and of the independent nation of Ghana. The Port of Tema is the primary seaport of Ghana and one of the major cargo ports of West Africa. Corruption has long challenged port business worldwide and the Port of Tema in Ghana is no exception. Although Ghana is one of the most stable democracies on the African continent with presidential elections held every four years, many Ghanaians share a perception of a high prevalence of corruption in their society. Partly therefore, as of 2022, Ghana ranks 72nd in the Corruption Perception Index (https://www.transparency.org/en/cpi/2022). In the context of this article, we define corruption as opaque transactions, where individuals abuse their power for private gain. Such activities in turn impede socioeconomic growth and obstruct port efficiency, not least when expressed in the forms of individualized, unpredictable, and capricious demands characterized by some researchers as “arbitrary corruption” (Gelbrich et al. 2016; Uhlenbruck et al. 2006; Rodriguez et al. 2005).

National policies, international regulations, and private sector interests frame the operations at all ports. As a centrally planned deep-water port and town area, the Port of Tema was first envisioned during the final decades of British colonial rule with construction initialized in 1954 (Doxiades Associates 1962) and made operational in 1962, five years after Ghana gained national independence (Chalfin 2010). Over the following decades, Tema underwent periods of expansionary investments as well as periods of relative stagnancy (Chalfin 2009). Since the late 1990s, the port has attained progressively higher significance for the national economy. This has been supported in part by national trade policy changes of the Ghana Trade and Investment Gateway Project from 1999 (World Bank 2013; Ackah and Aryeetey 2012), which aimed to make Ghana a hub for trade in West Africa. An overall political desire drove these reforms to use privatization of state-owned enterprises as a way to improve the national finances. This national political pursuit entailed both divestiture of monopolistic state companies and liberalization of whole business sectors to stimulate enhanced efficiency through competition (Appiah-Kubi 2001).

In recent years, the port underwent significant expansion to increase its capacity from 12 to 20 berthing spots, with depths ranging between 8.2 to 16 m. The new spots with the deepest depths allow some of the world’s largest ocean-going cargo vessels to take berth. From the 1960s through the early 2000s, the limited capacity of berthing spots made the Port of Tema prone to congestion, which meant that commercial vessels frequently had to wait at anchor off the coast for days or weeks before the port authorities assigned them a berthing spot. Consequently, the competition for timely berthing spots was fierce among shipping agencies.

The objective of the push towards transforming the Port of Tema into a landlord port was the complete divestiture of the public institution Ghana Ports and Harbours Authority (GPHA) from stevedoring (World Bank 2013; Ameyaw-Akumfi 2006). The liberalization in the 2000s and 2010s came with politically decreed increases in the total number of licenses to be granted to privately owned stevedoring and shore handling companies. Over the years, the stevedoring business at the Port of Tema had grown from originally one state-owned Stevedoring Department to three companies (GPHA, Atlantic Port Services, Speed Line). In 2002, GPHA licensed the 6 additional companies, Express Maritime Services, Golden Gate Services, Carl Tiedemann Stevedoring, Odart Stevedoring, Dashwood Stevedoring, and Fountain View. Later, in 2017, the number of licensed companies was increased to twenty, and as of 2023, 22 stevedoring companies are registered at the port (GHPA 2023).

In the late 1960s, many disputes and delays led local public and private stakeholders to take the initiative to conduct the so-called berthing meetings informally, and around 1986, the same year that the Provisional National Defence Council (PNDC) of Ghana established GPHA, they established the Berthing Meeting Association of Tema (BERMA) as its formal joint welfare association. The berthing meetings have since continued for close to 40 years. Prior to the COVID-19 pandemic, typically 50–60 representatives of various public and private stakeholders met for 2–3 h every Monday, Wednesday, and Friday in a conference room at the Port of Tema. Due to public health concerns following the onset of the pandemic, the Wednesday meetings have since March 2020 been indefinitely suspended, just as the attendance throughout 2021 was softly capped at 25 persons, although in practice typically 30–40 people still joined each meeting.

BERMA has two components, a business component, where participants negotiate and decide on berths and work assignments, and a welfare component, which offers the members various kinds of support and privileges. The chairman of the berthing meetings is a representative from the Tema Harbour Master’s office together with one or two colleagues from the port signal station, both operating under the authority of GPHA. These uniformed maritime officials sit at a separate table and attend to the practical assignments of vessels to berths and dockside work crews to vessels. Meanwhile, elected BERMA members manage the berthing meeting agenda, general conference room decorum, and ongoing membership fundraising. The official purpose of the fundraising at the meetings is a social security network to support attending members and their dependents in case of health-related issues (accidents, illness, death), major life events (marriage, retirement, funerals), educational membership seminars, and an annual party (BERMA 2016).

The formal purpose of the berthing meetings is to publicize the distribution of berthing spots for incoming commercial vessels as well as the related cargo work for the local stevedoring and shore handling companies. Among the commercial participants are representatives of both large and small shipping lines and agencies, stevedores, dockside labor, equipment rental companies, environmental service companies, and ship chandlers, who deliver supplies for vessel crews. Ghanaian public stakeholders include representatives of port institutions such as GPHA, Ghana Shippers’ Authority, Ghana Maritime Authority, Ghana Immigration Service, the GPHA Fire and Safety Department, and the Harbour Police. Prior to each meeting, the Tema Harbour Master’s outfit has assigned berths to vessels in the order in which they arrived. The few exceptions are based on established priorities, such as containers, fresh fruit produce, and ro-ro vessels, which carry vehicles. Secondly, GPHA has overseen the distribution of stevedoring jobs according to the running tally kept with the port authorities. GPHA records this information in digital spreadsheets, which they share electronically with the relevant stakeholders.

During the morning preceding each berthing meeting, representatives of two local Tema stevedoring companies meet with the representative of the Harbour Master’s outfit to assign what is called the “pre-berthing allocation” of stevedoring crews among vessels about to take berth. This task rotates among all licensed private stevedoring companies on a fixed schedule every two weeks, so that each company gets to partake in the practical division of dockside labor among all the eligible private stevedoring companies. GPHA still reserves 25% of the total vessel cargo work for its own Stevedoring Department, but the current 22 private companies split the remaining 75% of work. The Monitoring and Statistics Department of GPHA keeps a running tally on a digital Microsoft Excel® spreadsheet based on how many tons of cargo each stevedoring company has handled each month, and the company with the least work done so far gains the highest rank for obtaining future job assignments. The explicit purpose of this procedure is to ensure an equal share of the total work for each stevedoring company. Our interviewees from both the operational level and the GPHA corporate headquarters in Tema (GPHA 2020 interview) confirm this.

## Theory

By combining existing literature on network theory, trust literature, and studies on humor, we investigate how the physical berthing meetings at the Port of Tema in Ghana ameliorate the negative consequences of breaches in business ethics. First, the distinction between weak and strong ties as defined by Granovetter in 1973 provides a framework for analyzing the constellation of participants. Granovetter (1973) defined weak ties as relations between individuals from different groups and strong ties as relations between members of the same group, village, or company. Granovetter argued that people sharing strong ties already have similar characteristics and information, which limit change and innovation, whereas weak ties offer new information and options. According to the quantitative content analysis of Castaldo et al. (2010), the focus on relations within the same company or organization or between company representatives and external clients is a primary concern of most of the prominent articles on trust in market relationships. However, we have not found studies about trust between commercial rivals and trust in complex settings of multiple public and private stakeholders. Additionally, while some studies analyze efforts to repair firm-stakeholder relations after breaches of trust (Brown et al. 2016; Goodstein & Butterfield 2010), we have found the literature sparse regarding situations where business stakeholders continuously need to work together, despite a mutual history of deceitful business practices and outright corruption, in other words, situations where each participant still has reasons to question the ongoing ethical conduct of their peers and rivals.

The literature on trust and distrust enables us to distinguish between achieving trust and managing distrust. The literature on institutional trust in Ghana is quite large. Godefroidt et al. (2017) argue that institutional trust in Ghana reflects how Ghanaian nationals distrust political actors rather than the democratic system. Bob-Miliar and Lauterbach (2021) nuance this argument by finding that Ghanaians express trust in the political system more than in any political party, and in single political parties more than in the government. They identify “corruption” as one of the key determinants of such distrust among Ghanaians (ibid, p. 87). In a similar vein, Adelopo and Rufai (2018) point to the creation and maintenance of “trust” as the key component in successful anti-corruption efforts, not least in governmental institutions. Still, Lawson (2009) highlights the perpetual challenges that anti-corruption reforms face in neo-patrimonial African contexts, where political powers frequently use corruption charges against their opposition, which limit the impact of reforms.

Wicks and Berman (2004) present some theoretical considerations for business settings where institutional, sociocultural, and industry-based trust support mechanisms are lacking, and they include many African nations as examples. Recent articles about business ethics in Africa have emphasized opportunities and challenges in adopting approaches indigenous to different African countries and contexts (Adeleye et al 2020). In line with the articles about local concepts such as Ubuntu in South Africa (Pérezts et al. 2020; West 2014), this study brings this perspective into the maritime domain and highlights the integration of local insights and humor into strategies for dealing with challenges to the daily operation of the Port of Tema. Our aim has been to discuss the value of humor among local stakeholders for the port environment, which makes our findings applicable beyond Africa.

Theories about the effects of humor exist in several academic disciplines. Anthropologists, historians, sociologists, and psychologists have all contributed to understanding the role and uses of humor in various contexts (Romero & Pescosolido 2008; Carty and Musharbash 2008), yet for the present purposes, we rely mostly on the works of Fine and De Soucey (2005) for overall analysis of the role of humor in group identity reproduction and in handling potentially delicate topics, supplemented by Cann et al. (1999) and Morreall (1991) regarding the issue of how humor de-stresses. Based on analysis of multiple prior works, Fine and De Soucey argue that joking cultures serve to regulate social groups by (1) smoothing interactions between members, (2) shaping collective identities through cohesion, (3) drawing boundaries between the group and others, and (4) enacting informal social control. These four facets of humor in group settings are all in various ways evident in the ethnographic materials recorded from the berthing meetings in Tema, where an intentionally organized light-hearted atmosphere frames the overall serious livelihood pursuits that prompt everyone to attend.

Morreall (1991, p. 361–363) explains how humor allows people to stay in control of their emotions by engaging a larger perspective on situations that would otherwise overwhelm them with fear or anger, which are emotions that lead to loss of control and by extension to stress. Cann et al. 1999, p. 179, 187–188) combine experiments with regression analyses to argue that humor reduces anxiety and increases positive affect. However, they also emphasize the role of the sense of humor of individuals. We apply this concern with individual sense of humor to the role of a shared Ghanaian sense of humor drawing on references to common languages and culture, which reflects the predominantly Ghanaian maritime industry participation in the meetings.

## Methodology

Our study draws on ethnographic fieldwork, contemporary and historical materials related to the port, and related academic discussions. The ethnographic materials include 19 qualitative interviews and observations among the Port of Tema stakeholders in 2019–2022, five-month long social media WhatsApp conversations with operational level port workers during Covid19 lockdown in 2020, and attendance at 60 berthing meetings constituting an equivalent total of 25 weeks of port activity planning. Using anthropological methodology, we collected empirical data considering both physical settings (DeWalt & DeWalt 2011) and digitally mediated settings (Pink et al. 2016). The observations and interviews were part of a larger research project on the efficiency and public private capacity development funded by the Danish Foreign Ministry, where two Ghanaian scholars from business economics and maritime studies, respectively, and four Danish scholars from departments of anthropology, history of ideas, business, and history engaged in joint fieldwork at the Port of Tema.

Nobody formally takes minutes at the Tema berthing meetings. Each participant usually jots down their own hand-written notes in the margins or on the back of their personal copies of the day’s printed-out gazettes with lists of vessel information. Our own ethnographic note taking during the period of study has therefore served as the primary source to establish a detailed journal of the events and discussions of each day (Emerson et al. 2011). For our ethnographic data collection, we have paid special attention to the documentation of cases concerning demands for payment of fines and donations, as well as the documentation of significant instances of humorous expressions among attendees. We consider expressions significant where such gained the attention of the whole conference room. The registration of demands for fines and donations included notes on the sums demanded and reasons expressed, while omitting the names of the people involved in order to ensure anonymity. Contemporary and historical documents inform our analysis of the concrete operations and policies related to the Port of Tema, which we look at through the lens of academic literature on ports, trust, humor, corruption, digitalization, and politics relevant to the Ghanaian context.

The combination of materials allows us to explore the complexities of and various perspectives concerning what is at stake in the discussion of the role of humor at the berthing meetings. However, the dynamics of the meetings and the flow of the jokes and interactions in real time act as a constraint on the options for collecting more quantitative evidence. As observers of what are essentially business meetings, we took handwritten notes, but could not record or interrupt meetings to seek clarification. In specific cases, we sought clarification after the meetings by posing direct questions to individual attendees. Another limitation on the options for data collection was the highly dynamic atmosphere at the meetings, where several simultaneous, spontaneous conversations or joking comments among different subgroups constantly took place. Capturing anything more than the words of the main floor speaker at a given time, combined with occasionally the gist of audible subgroups’ in-joking comments, was not feasible.

## Findings: distrust in a politicized port business environment

We find that distrust among competitors at the Port of Tema derive from in-transparency, perceptions of politically motivated licensing practices, and the general acceptance of bribes as an effective way to affect decisions concerning daily operations. Recurrent incidents of ethical breaches and the overall politicized nature of both the past and contemporary business conditions at the Port of Tema have had a detrimental effect on the levels of trust exhibited between stakeholders working there. Most people working at the port know that many private companies have obtained their business licenses because of political networking and by throwing public support behind the parliamentary election campaign of the winning party. In the general case of Ghana, Appiah-Kubi (2001, p. 223) notes how the use of government divestiture programs as an instrument of political patronage and the characteristic lack of transparency surrounding these processes have inspired allegations of corruption. The overall topic of corruption has been a mainstay of Ghanaian journalism and public debate for decades (Hasty 2005). In recent years, the work of the Ghanaian investigative journalist, Anas Aremeyaw Anas, together with the efforts of other undercover reporters and whistle-blowers has fueled the attention to this topic. Our interviews with business actors in the contemporary Port of Tema echo similar concerns on the continued prevalence of corruption across Ghanaian society.

One example, our interviewees often mentioned how political intervention has caused the great expansion in the number of private stevedoring company licenses, where successive governments during the past decades have rewarded company licenses to some of their political donors. Political influence, as long-term national policies, politicized appointments of top-level administrative management, and politicized licensing have for a long time affected the business environment in Ghana, not just the Port of Tema. A number of scholars including Asunka (2017), Quartey (2007), Appiah-Kubi (2001), Bob-Milliar (2012), Lindberg (2010), and Ablo and Overå (2015) have discussed the topic of politicization in Ghana. Asunka’s 2017 article shows a link between voter loyalty to political parties and politicians’ tendencies to distribute tangible benefits to members of their clientele networks. Furthermore, Bob-Milliar (2012, p. 597) describes how factionalism within both the two main political parties, NPP (New Patriotic Party) and NDC (National Democratic Congress), has largely revolved around the obtainment of privileges and resources for each faction’s members since the 1992 return of democratic elections in Ghana.

Senior shipping agents of Tema described their work environment prior to the establishment of berthing meetings in the 1980s as defined by fierce competition and distrust. As one senior of the Tema shipping business explained, from the 1960s and through the 1980s, shipping agents would often resort to “unorthodox” methods in order to bring themselves ahead by sabotaging the work of their competitors. Agents paid bribes to politicians or to senior port management to make port officials ignore the first vessels already at anchorage and let the agent’s freshly arrived vessel take berth right away. The sabotaging agents would then often go so far as to contact their competitors’ principals to encourage them to switch over to their agents’ service by pointing out how the other agents had been inefficient compared to this agent by failing to secure their vessel a berthing spot quickly.

The operations manager of one of the stevedoring companies in Tema licensed in 2017 recounted in a 2019 interview how his former stevedoring company employer lost their license after the 2016 parliamentary elections. GPHA under the NDC government originally granted the former company’s owner the business license. However, when the NPP government took over, GPHA terminated rather than renewed this license. Instead, another NPP-supporting company took over the license, and hired him as operations manager for this new company. The interviewed stevedore had immediately proceeded to rehire everyone from his old stevedoring company crew, because they already constituted an experienced team ready to work. Therefore, the transition between the stevedoring companies was from his point of view more of a formality than a real setback in terms of dockside work efficiency. The stevedore operations manager considers 10 stevedoring companies enough to handle the current cargo needs of the port, but of course, he adds, everyone in this business needs to adapt to the present realities.

Because the GPHA makes most of the choices regarding the distribution of dockside work and issues temporary stevedoring company licenses subject to annual renewals, most private operators in the stevedoring business find long-term investments in equipment and labor risky. An important exception here are the Tema berths controlled by the Meridian Port Services (MPS) container terminal, which is a mostly independently run joint venture initially between the international shipping companies AP Møller-Maersk Terminals, Bolloré, and GPHA. Officially, GPHA renews port contractor licenses annually (GPHA 2019, pp. 61–62). However, among the license holders, there is an understanding that the licenses are for the four-year period of government tenure, which supports the argument that there is a general understanding that politics influence the GPHA and most port affairs—an understanding shared by GPHA officials and most other actors we interviewed concerning the port.

As shared by a business development manager at the Maritime Labour Ltd. Company in Tema in an interview conducted in 2020, most of the currently licensed private stevedoring companies in Tema lack not only their own total complement of equipment, but, frequently, also enough skilled labor. Instead, they rent equipment day-to-day from companies, such as his, which GPHA has licensed to provide this specialized service. The Maritime Labour Ltd. Company itself operates under an annually renewed GPHA-granted governmental business license, which the business development manager stated in the interview that he expects will be renewed as long as the current government is in power. Consequently, his own continued employment with this company depends on whether GPHA will renew the license under a new government.

Only twice, for short periods in January 2004 and again in January 2005, did the Port of Tema try to make the licensed private stevedoring companies compete freely with each other for work contracts. The first attempt was abruptly halted after a few weeks in January 2004 (GhanaWeb 2004). The next attempt was started from 1 January 2005, according to a Ghana News Agency article from 19 December 2004 (Modern Ghana 2004). However, according to the recollections of some of the affected local stevedores interviewed in 2019–2020, these periods of open competition quickly devolved into distrustful agitation among the stevedoring companies of Tema and resulted in allegations of favoritism and insider deals. Consequently, at the request of the private stevedoring companies themselves, the port soon went back to distributing the cargo work assignments according to fixed percentages of the total tonnage among all of the licensed companies. Regardless, the politics of the port continued to increase the number of stevedoring companies, which mirrors perceived opaque license criteria and profit distribution.

The ability to trust in the ethical, law-abiding conduct of one’s professional peers and competitors may constitute the ideal condition for doing business (Castaldo et al. 2010; Brien 1998). Veterans of the shipping business of Tema (2019–20 int.) have recounted tales of professional misconduct that reach back to the earliest years of the port itself. According to long-standing BERMA members, the main reason for initiating the Tema berthing meetings in the late 1960s was that widespread unorthodox and unfair competitive practices had resulted in tensions that had soured the general working conditions for many of the people earning their livelihood in the port. People saw each other as competitors rather than fellow stakeholders, everybody expected deceit from others, and no one would come to another’s aid.

According to interviews with senior stakeholders of the Port of Tema, the establishment of berthing meetings has led, over the past several decades, to a lessening of competitive enmity among both shipping agents and stevedores. Now, stakeholders of the businesses meet and sit together in person for the announcements of berthing arrangements, and, thereby, each can witness how the Tema Harbour Master’s outfit assigns vessels to berths in the order that they arrive, and how stevedoring jobs are allocated according to tallied quotas. Notably, at the meetings, attendees can request to have any deviations from these principles immediately addressed and clarified, because valid practical factors often do cause need for deviations. Such factors might include draft (depth of keel), LOA (length over all of vessel), type of ship, availability of adequate and appropriate equipment (both fixtures at specific berths and equipment capacity of specific stevedoring companies), and type of cargo and port conditions (such as tide and weather). Public discussion of these factors within the forum of the berthing meetings has removed much of the enmity among shipping agents as well as among stevedores. The reason is the fact that the representatives of the various businesses, when included in the discussions of valid practical factors, no longer needlessly suspect their rivals of secret foul play in their pursuits of timely berthing spots for their principals’ vessels or shares of the cargo work.

In addition to appreciation of these qualities of the physical berthing meetings, some interviewed berthing meeting attendees expressed strong concerns about the ramifications of a potential full conversion to electronic vessel booking systems at the Port of Tema, and voiced concerns about the idea of future abandonment of physical berthing meetings. For such a move, they felt, could inadvertently result in increased levels of enmity and distrust among professionals at the port. Because individual stakeholders sitting alone at their company office desk and receiving only digital notifications of upcoming berthing assignments, may too easily assume that corrupt practices rather than valid practical factors caused the deviations from the original plans and criteria. Without the personal negotiations at the actual berthing meetings, such a person would no longer have a direct way to enquire about the reasons for such events—nor the immediate support of a large number of their professional peers in cases of actual, unwarranted line skipping. Effectively, the berthing meeting stakeholders worry that a complete digital transformation of the port systems might once again result in reduced transparency and accountability. To the participants, the physical berthing meetings continue to enjoy popular support, because their multi-stakeholder watch-dog function supplements the online systems with an in-person transparency and a “checks-and-balances” function. Furthermore, face-to-face interactions and the pursuit of a humorous setting help foster a sense of a joint port stakeholder community and a more positive work atmosphere.

## Findings: humor builds community

The humorous practices of berthing meetings create a sense of community for competitors and colleagues alike through shared experience and the provision of a social security net. The participants’ endearing reference to the meetings as the “Parliament of the Port” refers to humorous practices at the meetings, but also to more serious principles of democratic values, which give a voice to all actors. These principles can be seen, first of all, in the firmly established meeting procedures, characterized by the formal way, in which all attending members make sure to address each other as “Honorable.” Such stylized forms of address mirror the traditional features of a national political parliament setting, a comparison also evident in the way in which the berthing members as a group tend to refer to themselves collectively as the “Honorable Members of the House.” Moreover, members and invited visitors perform a formal greeting, which entails that each individual person, as they enter the meeting room, loudly proclaims “BERMA!”, to which all others in the room in unison reply “Anchor!”. A person’s failure to announce their entry this way may result in a so-called funny fine. The involvement of all in this communal greeting and the satirized overall formality of titles and ways of addressing one another mirror aspects of a joking culture that in Fine and De Soucey’s (2005) terminology contributes to the definition of this social group as distinct from others.

The “funny fines” constitute a major and well-known practice at the berthing meetings as a shared rule set that keeps shifting and, therefore, serves more as a source of entertainment and fundraising than as an actual incentive for regulating behavior. Throughout each meeting, the formally elected “Whip” issues fines to individuals attending the meeting. Some funny fines are issued according to fixed criteria, such as a 1 Ghana Cedi^1^ lateness fine and a 2 Cedi fine of anyone whose phone rings or who answers their phone during a meeting. At Friday meetings, anyone who attends without wearing a maritime company shirt, uniform, or “African wear” style of clothing is likewise liable to pay a 5 Cedis fine. However, the creativity and humorous whims of the Whip in charge on any given day determine the specifics of most funny fines. Each fine usually constitutes a minor cash amount between 1 and 10 Cedis, or in some cases, the Whip simply demands that an offending member donates to The House an amount of their own choosing. Donations may for this reason vary significantly, typically within a range from 5 to 100 Cedis, because each donating member more or less freely chooses the amount of money to donate, depending on the context and the exerted level of peer pressure. Seeming arbitrariness, unfairness and unavoidability are key characteristics of the issuance of funny fines. For example, at one meeting, one of this article’s authors was fined 5 Cedis for whispering something to the person seated to the left of him—and then again, another 5 Cedi fine some twenty minutes later for not having said anything at all to the person seated to his right.

What makes the fines “funny” to the meeting attendees is the purposefully arbitrary and unfair reasons, for which they are issued, which both mimic and mock the perceived absurdity and unavoidability of unofficial “fines” or facility payments demanded of people across many parts of the wider Ghanaian society. The Whip can simply choose to fine someone for any kind of made-up reason, the sillier, the funnier. Sometimes, he issues fines by reference to a person’s physical appearance such as in the case of untied shoelaces, a new haircut, or a remarkable choice of clothing. At other times, he issues funny fines due to a person’s behavior, for instance: “Honourable [X], have you changed your seat? – Pay 5 for transit!” and “Honourable [X], you are enjoying the current too much – 5!”. In the previous example, the person sat close to one of the three air conditioners in the room. The duality of the business of distributing berths and stevedoring assignments by the Harbour Master’s outfit and of the welfare component creates very lively and, at times, rather chaotic interactions. Despite the fact that the Whip employs humor to maintain order and discipline by issuing “funny fines” left and right as in this example “Honourable [X] and [Y]; you are talking with each other without permission – two for 5!”, the participants still engage in many parallel exchanges of words around the room.

At other times, the Whip issues funny fines for reasons related to a person’s work at the port. For example: “Honourable [shipping agent], pay 5 for ‘Sakura’.” [Funny fine issued to the shipping agent of a recently arrived vessel named NM Sakura. ‘Sakura’ in the local Twi language means ‘a clean-shaven head’.]; “Honourable [shipping agent], your vessel is named MSC England, but it is registered in Liberia. For having dual citizenship, pay 10 Ghana Cedis!”; or “Honourable [shipping agent], please donate for allowing the virus to hold on to your vessels at China.” The last fine related to the COVID-19 virus that in late February 2020 caused massive delays in scheduled exports from China. The agent, therefore, was not in any way responsible for the rescheduling of arrival of two Maersk Line vessels for one week to one month later, respectively. Likewise, in the two preceding examples, nobody could truly blame the agents for neither the vessel naming conventions nor the nationality of registration chosen by the ship owners. The participating Ghanaians readily understood all of these jokes, and after the meetings, they enlightened us about the humorous connotations, demonstrating how the humorous atmosphere at the meetings depends on shared Ghanaian linguistic, cultural, and maritime frames of reference.

Finally, in addition to the individual funny fines, during each berthing meeting, the acting Whip typically announces one collective fundraising so-called exercise. This means that the Whip decides on a specific criterion and anybody who happens to meet it will need to pay a fine. Examples of exercises include a 2 Cedi fine for not wearing a Tema harbor access permit ID badge around their neck and when the Whip announced: “If you are wearing white, you are challenging the color of the Chairman [i.e., the white maritime uniforms of the Harbour Master’s office]. Each of you come donate 2 Cedis.” And a final example of a 5 Cedi fine for having a beard. The occasion for this exercise was stated by the Whip as “support for our Muslim friends who are about to start their Ramadan.” The last example indicates that the BERMA members respect and accept their diverse religious background, which resonates with Godefroidt et al. (2017), when they conclude that religion and ethnicity are only marginally influential on institutional trust in Ghana.

The members’ overall expectation of fairness balances the intentional unfairness of the funny fines, because, over time, they expect these funny fines to hit everybody more or less equally hard. Consequently, there is no single “correct” behavior that will enable someone to avoid fines as illustrated by this remark “Honourable [X], for escaping my attention and not being fined today: pay 5 Cedis”. This expectation towards equality of contributions through funny fines also means that neither the person in the position of BERMA President nor the Whip themselves remain safe from being fined. For example, when the President says to the Whip: “Honourable Whip, by my presidential power I fine you 10 Cedis for being a very effective Whip today.” Or the Whip states “Honourable President, you are too happy – 5 Cedis!”. The experience of fairness in the distribution of funny fines reflects the trust in the democratic qualities of the institution of the berthing meetings, which runs counter to the attendees’ experience and expectations of the democratic standards of the national parliamentary system. “Funny fines” and penalties, which fail to regulate behavior, function as the primary method to fundraise for the welfare component of the Tema Berthing Meeting Association and therefore the incentive for misbehaving might lie in the increase of welfare funds.

The use of the fines builds community, because the collected money is used for a shared good in the shape of a social safety net—and what is left over for an annual party for all members and their families and friends. As opposed to hidden facility payments and other fines and donations in the surrounding society, BERMA members consider it crucial that members pay all funny fines in cash, immediately, and for all of them to witness this with their own eyes. It has for this reason become established procedure that the entire formal meeting agenda and discussion is placed on hold whenever a funny fine is issued, only to be continued after cash payment of the fine has been made and confirmed. The Whip leaves the collected cash fully visible in a pile on the central table in front of himself throughout the entire meeting. At the closing of the meeting, the person elected to the role of Treasurer tallies and accounts for the money, and then deposits it in the bank account of the association. The collected “funny fines” and donations all officially go to finance the welfare component of BERMA, and the weekly aggregated sums collected during the 25-week period of study have ranged between about 800 and 1.100 Cedis. Payouts of benefits involve predefined sums of 400 Cedis for each marrying or retiring group member and 1.000 Cedis to the closest next of kin in cases of a member’s death (BERMA 2016, p. 9). Additional fundraising during the berthing meetings supplements the fixed amounts. The visibility of the money collection during meetings and the direct use for the common good of the shared BERMA community differ significantly from the experiences outside the meetings, which has led to Ghanaians consistently perceiving their own society as corrupt according to the Corruption Perception Index referenced earlier.

Beyond the funding of tangible social security benefits to the group members, the funny fines serve to build group cohesion through shared experiences. Everybody attending the meetings get to, recurrently, feel the uncomfortable spotlight placed on themselves, when the acting Whip of the day for whatever contrived reason singles them out for a funny fine punishment. Because of this common experience, everybody can in turn relate emphatically to the moments when others around them, similarly, receive fines. Regardless of whether those persons may be colleagues or competitors outside of the berthing meeting room, at the berthing meetings, everybody are “Honorable,” and everybody knows that they themselves may at any moment become either the audience of a joke or the butt of one.

Furthermore, humor works as a way to address sensitive topics such as various forms of corruption, bribery, and facility payments and business dealings, which directly affect stakeholders at the port. As Fine and De Soucey point out, in a joking group culture “the joker cannot be called out on the implication of his or her remarks, because ostensibly there are no implications” (2005, p. 17). This way, members can frame and discuss topics of misbehavior leading to unfair or preferential treatments of others in a way that avoids direct confrontations or accusations against named parties. Throughout the meetings, the business-related announcements of the distributions of berths for vessels and work assignments for stevedores and shore handlers become publicly visible and open for discussion within a humorous collegial setting, rather than purely the electronically announced outcomes of closed-door negotiations. Berthing meeting attendees actively use humor to establish their own community in order to manage the perceived unfair and opaque activities, which they encounter and to which they fall victim in their everyday work.

## Discussion

The reluctance to give up on regular physical berthing meetings, despite readily available digital information, and despite personal monetary costs involved in attending each meeting, prompted us to search for potential analytical answers within social scientific theory. Our findings about the prevalence of mutual distrust among actors from politicians to dockworkers involved in port operations in Ghana and the seemingly absurd and time-consuming use of humor can explain the continued attendance at the physical berthing meetings. In the following, we discuss how. In Ghana, port operations face widespread distrust based on both past and recent experiences of corruption and political favoritism (GhanaWeb 2017a; Aryee 2016). Ghanaians are quick to describe their politicians and society as corrupt; nevertheless, it is difficult to gauge if some of the practices reflect their colonial past as concluded by Sardan (1999) and Wanasika et al. (2011) and, further, encouraged by the expectations of Europeans as described by an APM Terminals compliance officer (Interview 2017).

The present position of GPHA corporate management in Tema is that the physical berthing meetings at the port constitute an outdated format. Consequently, once GPHA’s own digital terminal operating system is fully functional, GPHA expects to withdraw their formal support for the in-person berthing meetings as an official platform for conducting business at the Port of Tema (GPHA interview 2020).

In September 2017, the Ghanaian Vice President and the Ghana Ports and Harbours Authority (GPHA) lauded the implementation of a paperless port transaction system as the end to an era of corruption, delays, and impeded efficiency in Ghana (GhanaWeb 2017b). The Ghanaian authorities announced that they expected that the introduction of digital systems will eliminate the human interface, which they blame for various corrupt practices. Presumably, an automated digital system for vessel berth assignments and stevedore cargo work tallying would provide an impartial distribution, which ought to assuage port stakeholders’ concerns caused by their own experiences of past manual malpractices (Marful and Brako 2016). As it is, digital systems are already widely in use at the Port of Tema, and the berthing meetings revolve around printed gazettes from some of these very systems. The official registration of vessels and the assignment of berthing spots and stevedoring jobs at the Port of Tema take place on digital platforms. As of 2022, GPHA uses the Unity® system for vessel bookings, and an Excel® spreadsheet solution for the stevedoring jobs. The Unity system linked to the GCNet® and Westblue® systems for cargo processing until the end of May 2020, when the UNIPASS-ICUMS® system replaced the latter two systems. The information is readily available to all the relevant stakeholders through circulated e-mails and logins to online databases.

Most operational level port users in Tema are (as of 2022) members of the same WhatsApp social media group organized by BERMA, as well as in contact with one another through telephones. However, the operational level stakeholders of Tema interviewed in 2019–2020 did not share the domestic and international official optimism towards the benefits of digital systems and expressed strong desires to continue the regular physical meetings. They remain reluctant to abandon the physical meetings and, exclusively, rely on the digital information accessible at their company offices and through smart phones. Contrary to the officially voiced expectations, the experiences at the port rather align with studies by Al-Shbail and Aman (2018) and Basyal et al. (2018) that show how e-government may not necessarily curb corruption. Instead, as argued by Gunz and Thorne (2020), Brink et al. (2019), and Johnson (2015), the adoption of digital solutions may give rise to new ethical problems due to loss of transparency and abdication of responsibility once human data inputs are intermingled with digital systems and no longer subject to direct person-to-person scrutiny. The findings of this article support these arguments, as they indicate that the continued local demand for physical meetings among competitors reflects a particular need for transparency in the port stakeholder community, which shares an interest in efficient port operations amid a highly politicized business environment. From this perspective, humor and transparency at on-site berthing meetings appear as central tools for the management of distrust at the operational level of the Port of Tema and constitute qualities that are not currently replicable in digital port administration systems.

The berthing meetings at the Port of Tema draw employees from all port businesses and relevant public offices including competitors, lending these meetings at least some similarities to the forms of multi-stakeholder initiatives discussed by Rasche (2012), whose work on loose and tight stakeholder couplings expands on Granovetter’s work. All meeting participants are Ghanaian nationals, almost without exception, even though they qualify as weak ties, because they work for different companies and institutions. Therefore, we find that this reflects the local interest in smooth port operations for those who depend on the port as their daily workplace, as compared to foreigners who can choose other ports and Ghanaian politicians who have many other sources of income. However, unlike the internationally linked stakeholder dynamics emphasized in Rasche’s work, the participants in the Tema berthing meetings are almost all Ghanaian nationals whether employed by a foreign investor, shipping line, or a local stevedoring company. Following Granovetter’s theory, the Ghanaian employees of different companies may be competitors, but they also share an interest in keeping distrust among one another at a level that maintains a bearable practical work environment and strengthens the overall status of the Port of Tema.

Because of the rich income opportunities at the port, breaches of business ethics committed by powerful foreign and national investors and politically connected stakeholders continue to create obstacles that cause disruptions to the regular flow of port operations. The ramifications of the preferential treatments and irregularities directly affect the people working at the port on a daily basis and made them create the berthing meetings in order to offer a space to negotiate how to cope with and possibly lessen the negative consequences to daily operations of distrust sown by corrupt practices on all levels. In terms of Wick and Berman’s (2004) theoretical framework, the berthing meetings can be taken to constitute an example of a case where local industry stakeholders join forces in an attempt to reduce distrust in a setting where both institutional and sociocultural trust levels are very low. Moreover, almost without exception, all meeting participants are Ghanaian nationals even though they qualify as weak ties according to Granovetter (1973), because they work for different companies and institutions. Therefore, we find that this reflects the local interest in smooth port operations for those who depend on the port as their daily workplace, as compared to foreigners who can choose other ports and Ghanaian politicians who have many other sources of income.

Moreover, our findings indicate that the predominantly Ghanaian participants regardless of employer share a common sense of humor, insights into the political structures of Ghana, and a perspective on Ghanaian society viewed from the port, which provides a mutual frame of reference for the expressions of humor. Hampes (1999) discusses the correlation between trust as intimacy and humor based on a Trust versus Mistrust Scale of Measures of Psychosocial Development, Coping Humor Scale, Multidimensional Sense of Humor Scale, and Situational Humor response questionnaire distributed among 89 subjects. He concludes that “a sense of trust” comes before “a sense of humour.” Our focus on humor among business competitors problematizes his conclusion, because we in our empirical observations see humor as a way to manage inherent distrust. The berthing meetings provide a space for “weak ties” regarding professions and employment to find common ground through their shared backgrounds.

The humor used at the berthing meetings allows participants to cope with their common experiences of arbitrariness and unfairness at the port and in the Ghanaian political system, which it is not readily within their power to change. This matches the Kujala et al. (2016) interpretations of cases where distrust increased trust, because of intra-organizational fragmentation, and where the critique of established practices in turn promoted improved ethical organizational behavior. We argue that physical presence at the berthing meetings offers a shared experience of transparency, where humor serves to create a sense of collective port user identity. Furthermore, humor functions as a strategy to relieve stress and as a tool for confronting difficult issues. Here we see a clear case of Fine and De Soucey’s (2005) argument that humor promotes collective group identity and makes interaction between different and competing strata of the collective smoother. In this case, the collective group identity builds on a critique of unofficial fines and penalties in Ghanaian society, which the members perceive as arbitrary, because they do not conform to Sampath et al. 2018, (p 745) definition that fines should consistently penalize misconduct and deviations from social norms.

The shared jokes fuel the collective identity of the BERMA members by enabling an agreeable work climate and providing opportunities to de-stress, as commented in 2020 by the Chief Whip: “A lot of people come and have some fun to release some stress.” The participants refer to the meetings as de-stressing, because of the exchange of jokes and various comical rituals such as the “funny fines.” Studies by Morreall (1991) and by Cann et al. (1999) support this experience of the effect of the meetings by the participants. Humor induces concrete feelings of relaxation and reduces stress levels at berthing meetings, but it also infuses the overall conceptualization and organization of the meetings. We found that the humorous framing and the transparency surrounding the collection of “funny fines” serve as (1) a deliberate effort to contrast the berthing meetings to the conditions characterizing many parts of the outside society and (2) a reflection of a shared wish for a well-functioning port.

Whereas individuals covertly demand facility payments in other settings, the berthing meetings transform such procedures into overt and humorous parody mocking their original referents. The exaggerated ceremonious formalities of the “Parliament of the Port” make fun of the perceived aloofness of a powerful, yet distant and reality-detached national parliament. The humor of the “funny fines” works by exaggerating the perceived unfairness of general facility payments through the justification of each fine with absurd reasons that a victim has no chance to avoid. Many outside factors still impede efficiency and cause frustrations among people whose livelihoods depend on the Port of Tema. However, the berthing meetings have come to serve as a venue for people to voice their frustrations, de-stress through jokes, and join colleagues and competitors to mitigate some of the negative effects of repeated breaches of business ethics on their shared work environment.

## Conclusion: managing distrust

We conclude that the berthing meetings in Tema have gradually grown to serve as a pragmatic local stakeholder adaptation to the challenges posed by universally perceived politicized, opaque, and corrupt business practices of the environment of the Port of Tema and beyond. We have argued that the berthing meetings are not so much a matter of building trust, as it is a locally designed mechanism for keeping distrust at a manageable level, because Ghana is a country with low general trust (Godefroidt et al 2017). The main stake at the berthing meetings is not trust, but rather access to information, ability to witness decision processes, and room for the negotiation of shared concerns at the operational level. We suggest that one of the key generators of community among otherwise disparate and competitively opposed stakeholders is the use of humor as a strategy in the organization of the berthing meetings and the conduct of the participants. We discussed how expressions of humor at the berthing meetings serve as a key element for the reproduction of a sense of shared community and as a tool for the management of distrust between competitively opposed port stakeholders. We found that the regular physical berthing meetings enable and support cross-company, cross-institutional networking, whereby they boost the internal industry transparency and capacity for stakeholder collaboration. Besides, they enable adaptability of operations at the Port of Tema business sector.

This article finds that the insistence on the continuation of the twice-weekly physical berthing meetings has little to do with a general distrust in the impartiality of the digital port systems alone. Instead, we would argue that the distrust among Port of Tema stakeholders is toward the human users of such digital systems. For this reason, dozens of public and private stakeholders each spend numerous working hours a week attending physical meetings at the port. Members of the Port of Tema community use the meetings that provide a positive and transparent venue for the management of distrust to keep tabs on each other and, collectively, perform checks and balances on business arrangements and political and administrative decisions that affect them all. The physical berthing meetings reproduce a multi-stakeholder port community between members. Consequently, the berthing meetings have reduced the opportunities for unscrupulous shipping industry actors to try to gain unfair competitive advantages by attempting to illegally influence port officials or in other ways to hamper the work of their competitors. Because the political interference continues, the berthing meetings still play a significant role in establishing what is perceived as a fair way to handle a situation where there is limited work for too many licensed stevedoring companies. Those who participate in these meetings see them as a way of ensuring transparency to counteract the influence of otherwise invisible corrupt practices.

This article highlights two promising areas for future research. One concerns pragmatic ways of coping with endemic conditions of distrust caused by factors beyond individual control. The other concerns the nexus between studies on humor and studies on trust regarding relations between competitively opposed business stakeholders. More knowledge is needed about the contribution of humor not only among colleagues, but also among rivals, to reduce the negative consequences of corruption on shared work environments.

## Data Availability

Data is available upon request.

## Footnotes

1 During the time of research in Spring 2020, the value of 1 Ghanaian Cedi (GHS) was equal to approximately 0.17 USD.

## Abbreviations

*GPHA*: Ghana Ports and Harbours Authority
*BERMA*: Berthing Meeting Association of Tema
*PNDC*: Provisional National Defence Council
*NPP*: New Patriotic Party
*NDC*: National Democratic Congress
*MPS*: Meridian Port Services
